# Comparative indoor and outdoor stability measurements of polymer based solar cells

**DOI:** 10.1038/s41598-017-01505-w

**Published:** 2017-05-02

**Authors:** Yiwei Zhang, Hunan Yi, Ahmed Iraqi, James Kingsley, Alastair Buckley, Tao Wang, David G. Lidzey

**Affiliations:** 1 Department of Physics and Astronomy, University of Sheffield, Sheffield, S3 7RH UK; 2 Department of Chemistry, University of Sheffield, Sheffield, S3 7HF UK; 3 Ossila Ltd, Kroto Innovation Centre, Broad Lane, Sheffield, S3 7HQ UK; 4 School of Materials Science and Engineering, Wuhan University of Technology, Wuhan, 430070 China

## Abstract

We report comparative indoor and outdoor stability testing of organic solar cells based on a blend between a donor-acceptor polyfluorene copolymer and a fullerene derivative. The outdoor testing was conducted for a period over 12,000 hours in Sheffield, England, with a Ts80 lifetime determined in excess of 10,000 hours (420 days). Indoor lifetime testing was performed on solar cells using a solar simulator under a constant irradiance of 1000 W/m^2^ for more than 650 hours. We show that under the conditions explored here, device degradation under the two sets of conditions is approximately dependent on the absorbed optical energy dose.

## Introduction

Organic semiconductors are being explored for applications as the active layer in solar cell devices^1–3^. Recent years have seen a growth in device power conversion efficiency (PCE), with efficiency values reported in excess of 10%^4–8^ for fullerene-containing organic photovoltaics (OPVs) and 12% for non-fullerene OPVs^9^. Such progress has come as a result of the design and synthesis of new materials together with the development of optimised material fabrication techniques. However, the achievement of high PCE is not the only impediment for the practical application of organic photovoltaic (OPV) devices, rather it is additionally necessary to reduce materials and manufacturing costs and extend operational lifetime. In recent years, a number of innovative fabrication techniques have been developed that are compatible with high volume, low-cost manufacture processes^10–13^. As a result of this, increased attention is now being paid to improving the operational stability of OPV devices^14–19^.

The operational lifetime of thin-film photovoltatic devices can be characterised by two different lifetimes, namely the T80 and Ts80 lifetime^20^. Here, the T80 lifetime is simply the time over which the device PCE reduces to 80% of its initial value. OPV devices however often undergo an initial period of relatively rapid reduction in their efficiency; a process known as ‘burn-in’. Following this, the reduction in efficiency then stabilises and drops at a slower, more linear rate. The exact identification of the end of burn-in period is not straight forward, but can often be identified by the onset of the period of linear reduction in device PCE. On identification of the end of burn-in, a second lifetime parameter can then be determined; namely the Ts80 lifetime. This is the time required for the device PCE to fall by 80% of its value defined at the end of burn-in.

The reduction in operational efficiency of OPVs over a range of time-scales (including burn-in) has been attributed to a combination of factors that can be initiated by the ingress of oxygen and water. These include oxidation or damage to device electrodes and oxidation of both donor and acceptor materials. The ingress of water can also induce aggregation of fullerenes or generate an insulating metal oxide interlayer at the interface between the active layer and the electrode that impedes charge extraction. The exposure of the active layer can also generate photo-oxidation reactions that either result in the formation of sub-gap states that cause additional recombination or reduce charge carrier mobility. Degradation can also result from thermal effects that drive morphological changes in polymer organization (disruption of π–π stacking)^21^, or induce aggregation or crystallization of the fullerene, limiting the OPV’s ability to successfully dissociate excitons. For a comprehensive discussion on degradation mechanisms that operate in OPV devices, we direct readers to a recent review^22^.

Extrapolated OPV Ts80 lifetimes in excess of 6.2 years have now been determined on the basis of indoor measurements performed using a solar simulator^23^. However it is necessary to explore device stability when used in outdoor conditions as laboratory-based accelerated lifetime tests rarely fully replicate all degradation processes to which a device operating under real-world conditions may experience. A number of recent outdoor experiments on encapsulated polymer:fullerene blend P3HT:PCBM OPVs have tracked device PCE over periods of thousands of hours^24^ and under different climatic conditions^25, 26^. Here, it has been shown that using suitable encapsulation schemes, devices can show remarkable stability, with the maximum power-point maintaining a value greater than 80% of its initial value over a period in excess of two years^27^.

It is clear however that outdoor-tests are time consuming and can take months or even years to complete. Unfortunately it is usually difficult to extrapolate between indoor and outdoor tests, as in general one measurement is not a simple acceleration of the others^28^. To address this issue researchers have started to provide a strategy to predict the lifetime of OPV devices based on indoor accelerated ageing tests. Here, Coraza *et al*.^20^ compared indoor and outdoor testing and concluded that for OPV modules fabricated using the same technique, devices tested using accelerated methods could undergo degradation in a period of days to months, whilst devices tested outdoors could have a lifetime of weeks to seasons. In their study, the outdoor and indoor lifetime testing was compared using a so called “o-diagram”, which plotted key parameters such as Ts80, providing a qualitative basis by which devices can be compared when aged under different conditions (e.g. light-soaking, dark-storage, high-humidity etc).

Most existing reports on outdoor stability tests of OPVs have focused on devices based on a blend of the polymer poly(3-hexylthiophene-2,5-diyl) (P3HT) with the fullerene phenyl-C61-butyric acid methyl ester (PCBM)^24–26, 29–35^. This OPV system has promising stability when tested outdoors, but devices suffer from a relatively low PCE. In recent years however, there have been reports of new donor-acceptor (D-A) type copolymers that have improved efficiency and stability under both indoor and outdoor testing^23, 36–40^. Indeed we have previously reported a study on the outdoor operational stability of OPVs based on a carbazole D-A polymer and found that devices had a Ts80 lifetime of around 6,000 hours^36^. This promising result has now encouraged us to explore other D-A polymers that have promising stability when tested indoors, and to determine whether this translates to enhanced stability under outdoor testing protocols.

In this study, we have explored the D-A copolymer poly[9,9-dioctylfluorene-4,7-alt-(5,6-bis (octyloxy)-4,7-di(2,2′-bithiophen-5-yl)benzo[c][1,2,5]thiadiazole)-5,5-diyl] (PFDT2BT-8). Our previous work has shown that this material can be blended with the fullerene acceptor PC_71_BM and then fabricated into OPV devices having a PCE of over 6%^41^. Studies exploring the exposure of thin-films of PFDT2BT-8 to AM1.5 radiation in the presence of air suggest this polymer should have promising long-term photo-oxidative stability^42^. To understand the extent to which stability measurements performed under laboratory conditions (subject to a constant irradiance of 1000 W/m^2^) can be related to outdoors testing in which there are significant fluctuations in temperature and light levels (with average intensity being approximately 10% of AM1.5 conditions), we have performed comparative stability measurements on encapsulated PFDT2BT-8:PC_71_BM OPVs. Admittedly there are differences between the testing conditions, as the devices tested outdoors had an additional level of encapsulation consisting of a nitrogen-filled metal can with a glass lid to protect them from oxygen and moisture. Interestingly however, we find that for both indoor and outdoor testing, the burn-in process and subsequent decay dynamics is approximately dependent on the energy dose received by the device. In both measurements, we identify a burn-in period, with indoor and outdoor measurements indicating Ts80 lifetimes of 530 and >10,000 hours respectively, with devices tested outdoors retaining a PCE > 3% after more than one years operation. This suggests that PFDT2BT-8 based OPV devices may have improved stability compared to those based on PCDTBT in which (using an identical methodology) we determined a drop in efficiency to 2.6% after one year’s operation^36^.

## Results and Discussion

**Figure 1:**
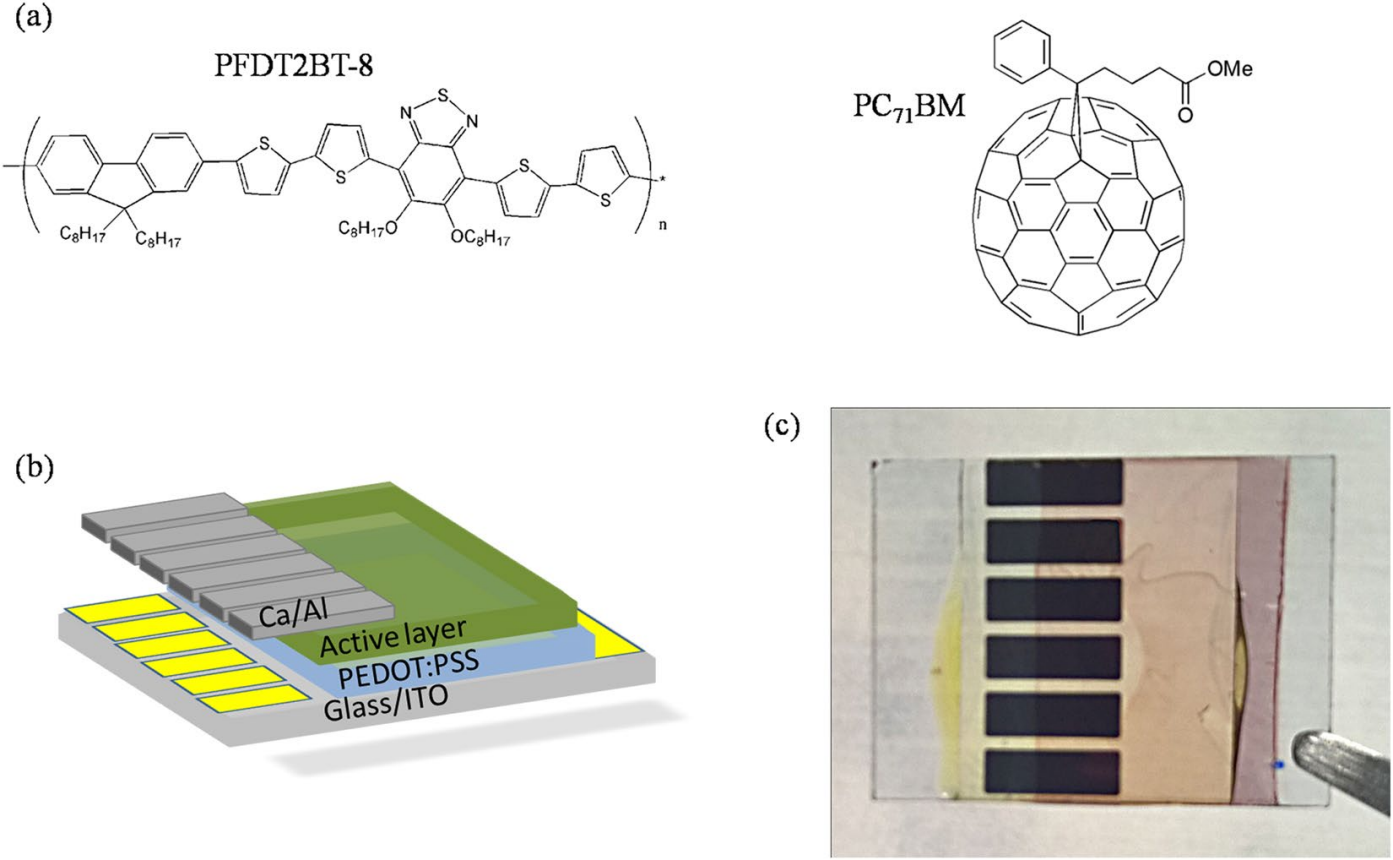
(**a**) Molecular structure of PFDT2BT-8 and PC_71_BM, (**b**) Schematic of device structure and (**c**) Image of a typical OPV device.

**Figure 2:**
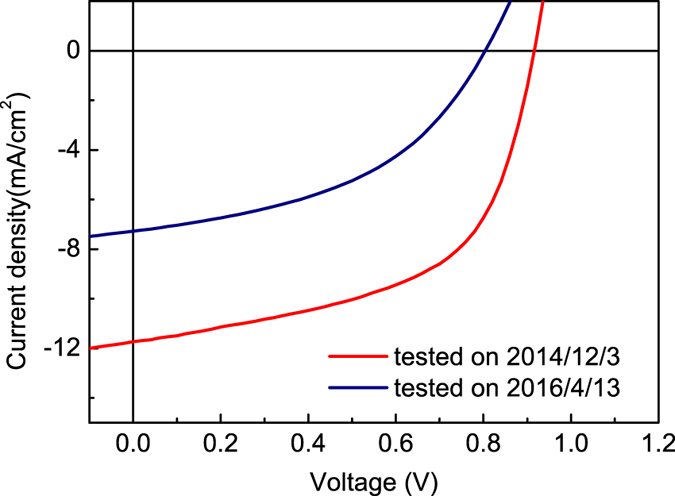
JV curves measured in laboratory at the beginning and the end of the testing period.

The molecular structure of PFDT2BT-8 and PC_71_BM is shown in Fig. 1(a). Figure 1(b) and (c) show a schematic of the device architectures studied together with an image of a typical OPV device before testing respectively. A *JV* curve of a typical device both at the start and end of outdoor testing period (vide-infra) is shown in Fig. 2. From this, we determine an initial device PCE of (5.9 ± 0.2) %.

Devices were tested at an outdoor lifetime testing facility located at the University of Sheffield (53°22′N 1°29′W). All devices were mounted on a test board that was sealed in an aluminium chamber having a toughened, glass lid transmitting light at wavelengths >350 nm. During testing, the chamber was filled with nitrogen at a slight overpressure. This chamber should be viewed as an additional encapsulation system that is necessary to directly protect devices from rain and humidity as well as limiting the ingress of oxygen to the active layer. A PXI-based multiplexer system (developed by Ossila Ltd. UK) was used to periodically measure key device metrics including power conversion efficiency (*PCE*), short circuit current (*J*_*sc*_), open circuit voltage (*V*_*oc*_) and fill factor (*FF*). Temperature sensors and silicon-photocells were also mounted on the test board next to the OPV device to record temperature inside the test chamber and the ambient irradiance. The sample chamber was mounted facing south at an angle of 30° to the horizon and was not shaded. The system was used to measure device metrics between 6 AM and 9 PM continuously, with each device measured at an interval of about 15 minutes. For the study reported here, four identical devices were located in the sample chamber, with each device comprising of 6 individual pixels. The devices were removed from the test chambers every two months and returned to the laboratory where they were re-measured using a calibrated Newport 92251A-1000 AM 1.5 solar simulator. Here, devices were tested using a calibrated aperture mask of known area (2.6 mm^2^).

Indoor lifetime testing was performed using an ATLAS Suntest CPS+ solar simulator with a 1500 W xenon lamp. Our previous measurements^37^ indicate that the lamp spectrum closely replicated the solar spectrum over the range 350–600 nm, corresponding to the absorption maxima of the polymer:fullerene active layer. Inside the test chamber, optical reflectors were used to create a uniform irradiation field of 1000 Wm^2^, with the ambient temperature and humidity inside the testing chamber being (38 ± 2) °C and (25 ± 5)% respectively. A multiplexor system was periodically used to measure device metrics, together with temperature and irradiation level. Here, our methods are in accord with ISOS-L-1 testing protocols^43^. Note however that devices tested inside the ATLAS Suntest system were not covered by an aperture mask, and so the device metrics recorded using this system are indicative only, with this technique only indicating trends in device efficiency. Furthermore, devices tested using the indoor system were not placed in a sample chamber, and thus the encapsulation glass fixed to the device surface with epoxy was their only protection against air and moisture. Clearly the degree of encapsulation differed between indoor and outdoor testing systems, and – as we discuss below – most likely contributed to the slightly enhanced degradation of devices tested indoors when normalised against the total quantity of absorbed optical radiation.

**Figure 3:**
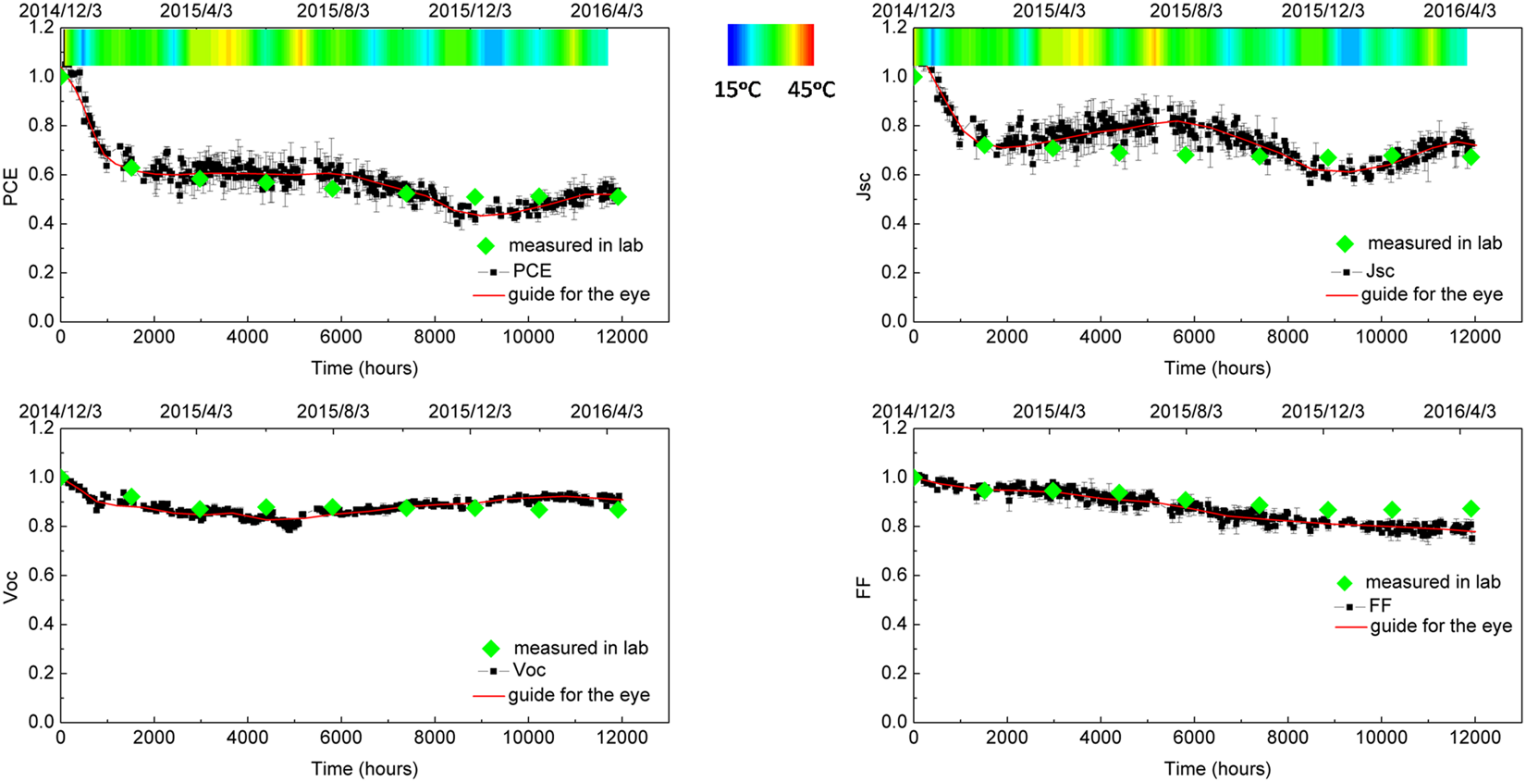
Evolution of the device metrics as a function of time (normalised to initial values) determined using the outdoor testing system.

We have analysed the data recorded outdoors after filtering using the following rules: (i) only the two central pixels were considered in the analysis as our previous study indicated that these pixels have the best stability as they suffer from less edge ingress effects, (ii) only data collected under irradiance between 500 and 1100 W/m^2^ was included (data collected during night or under cloudy conditions was removed), and (iii) data points in which device efficiency was 50% greater or smaller than adjacent data points were also excluded as can occur with rapidly fluctuating irradiance levels (for example from passing clouds) that prevent accurate normalisation. The measured irradiance was then used to scale measured *J*_*sc*_ to AM1.5 conditions. This value of *J*_*sc*_ was then used to calculate device PCE (assuming AM1.5 irradiation). Values of scaled *J*_*sc*_, *PCE*, *V*_*oc*_ and *FF* are shown in Fig. 3 as a function of time, with data normalised to initial values determined at *t* = 0. For comparison, we also plot the same parameters (using solid data-points) recorded when the devices were periodically returned to the laboratory for additional testing. A colour scale-bar is included at the top of the figure indicating the temperature within the testing chamber.

**Figure 4:**
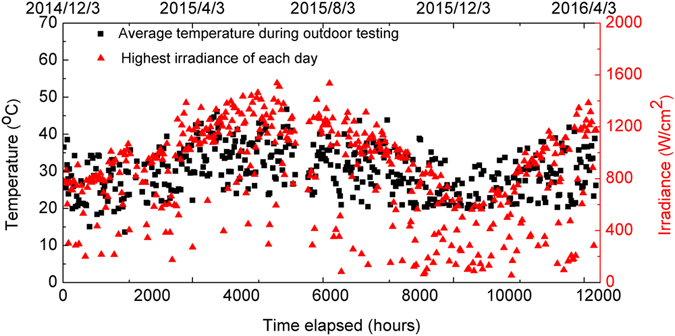
Seasonal variation of temperature and irradiance level.

In Fig. 3, it can be seen that during outdoor testing, device PCE dropped by around 38% during the early stages of the experiment. This fast degradation process is known as burn-in, and in this case slowed significantly around t ~1450 hours. Here, the turning point at the end of burn-in point is defined as Ts, with device efficiency at this point being Es. Using this, we calculate Ts80 as being the time over which device efficiency subsequently decays to 80% of Es^28, 44^. When device testing is performed under well-controlled, laboratory-conditions, the PCE usually drops in a steady, linear fashion in the period following burn-in. It can be seen in Fig. 3 however that there is considerable complexity in PCE determined in the outdoor tests as a result of seasonal temperature variations. Here, we find that most of this variation results from fluctuation in device *J*_*sc*_ with *V*_*oc*_ stabilised post burn-in and *FF* reducing at a slow but constant rate. Interestingly falls in *FF* appear compensated by an increase in *V*_*oc*_ at long times that help contribute to a partial recovery in device PCE. Note that the variation in the *J*_*sc*_ is most easily explained by the positive correlation between charge carrier mobility and temperature in organic semiconductor materials^45^. This can be confirmed by reference to the colour scale-bar and data shown in Fig. 4, it can be seen that the slow increase in *J*_*sc*_ at t > 3,000 h is positively correlated with the increased temperature measured inside the device testing chamber. We note that on the hottest summer day, the peak temperature in the test chamber briefly reached 70 °C. It is unlikely that this raised temperature had a detrimental effect on active-layer morphology and hence device efficiency. Firstly, we note that thermal annealing around this temperature in OPVs based on related materials is often used to generate small increases in device efficiency through the removal of residual casting solvent^21^. Secondly, our (unpublished) measurements on materials in the PFDT2BT-8 family suggest that the Tg of such materials (and their blends with fullerene acceptors) are around (120 ± 10)°C. Thus as the temperature in the testing chamber was at all points lower than the Tg of the PFDT2BT-8:PC_70_BM blend, we do not expect significant thermally-induced changes in the morphology of the active layer.

The effect of changing external temperature during outdoor testing can be evidenced by the periodic indoor-testing measurements which were performed at a quasi-constant temperature of ~25 °C (note the Newport Solar simulator runs at a lower temperature than the enclosed ATLAS Suntest system). The results of such measurements are plotted using solid data-points in Fig. 3, where it can be seen that device metrics undergo negligible seasonal dependent fluctuation over the period post burn-in. For completeness, the laboratory-recorded device metrics (averaged over 6 pixels from 3 individual devices) is tabulated in Table 1.

**Table 1:** Devices metrics measured in laboratory for devices undergoing outdoor lifetime testing.

Date (D/M/Y)	Elapsed time (hours)	*PCE* (%)	*J*_*sc*_ (mA/cm^2^)	*FF* (%)	*V*_*oc*_ (V)
03/12/2014	t = 0	5.9 ± 0.2	−9.7 ± 0.2	66 ± 1.5	0.92 ± 0.01
04/2/2015	t = 1512	3.8 ± 0.1	−8.6 ± 0.3	53 ± 0.6	0.84 ± 0.01
06/4/2015	t = 2976	3.5 ± 0.2	−8.3 ± 0.1	53 ± 1.2	0.80 ± 0.01
05/6/2015	t = 4392	3.4 ± 0.1	−8.1 ± 0.2	52 ± 0.9	0.80 ± 0.01
12/8/2015	t = 5808	3.3 ± 0.1	−8.0 ± 0.3	51 ± 0.8	0.80 ± 0.01
07/10/2015	t = 7392	3.2 ± 0.1	−7.9 ± 0.1	50 ± 1.0	0.80 ± 0.01
07/12/2015	t = 8856	3.1 ± 0.1	−7.9 ± 0.2	49 ± 0.8	0.80 ± 0.01
02/2/2016	t = 10224	3.1 ± 0.1	−8.0 ± 0.2	49 ± 0.5	0.80 ± 0.01
13/4/2016	t = 11912	3.1 ± 0.1	−7.9 ± 0.1	49 ± 0.6	0.80 ± 0.01

We find that after 12,000 hours, the devices retained approximately 52% of their initial efficiency and 83% of their efficiency at the end of the burn-in process. We determine that the Ts80 lifetime of our devices is in excess of 10,000 hours. Note that in our estimation of Ts80 lifetime, the PCE actually dropped to a value below 80% of its efficiency post burn-in value (after 9,000 hours), however this coincided with the UK winter. This efficiency subsequently recovered to a level above 80% of its post burn-in level when the weather became warmer at the start of spring-time (around ~11,000 hours – see Fig. 3).

We have performed a multi-factor polynomial regression fit using JMP software on the outdoors lifetime data (device metrics, temperature and irradiance) presented in Figs 3 and 4. This is plotted in Supplementary Information Figure S1. Such type of regression analysis is commonly used to sort through large data-sets and identify statistically significant correlations between seemingly unconnected parameters. For example, the analysis can be used to look for the effect of changing one parameter (e.g. temperature) between some set limits on a second parameter (e.g. fill-factor) given that all other parameters are held constant (e.g. illumination level).

Our analysis offers the following findings. Firstly, we find that device PCE increases significantly with temperature until around 40 °C after which it falls a little. This is likely to be related to a mobility enhancement (reflected by an increase in *J*_*sc*_) between 0 and 40 °C, with the fall in PCE at higher temperatures related to some different (unknown) mechanism. The device FF increases linearly with temperature; an effect that is also likely explained by a reduction in series resistance as bulk mobility in the device rises. The device *V*_*oc*_ seems to have a weaker temperature dependence and reduces at temperatures greater than 30 °C. Secondly, we find that the normalised PCE falls with increasing irradiance until a plateau is reached at around 600 W/m^2^. This effect possibly results from increasing non-radiative exciton quenching from free-charges within the device^46–48^. It has been previously shown that some irradiation induced defects can be “healed” by relaxation in the dark^49^. However a statistical analysis of our data suggests that there is no correlation between device metrics and the time of day at which they were recorded. This finding indicates that there is no apparent recovery of device performance overnight.

**Figure 5:**
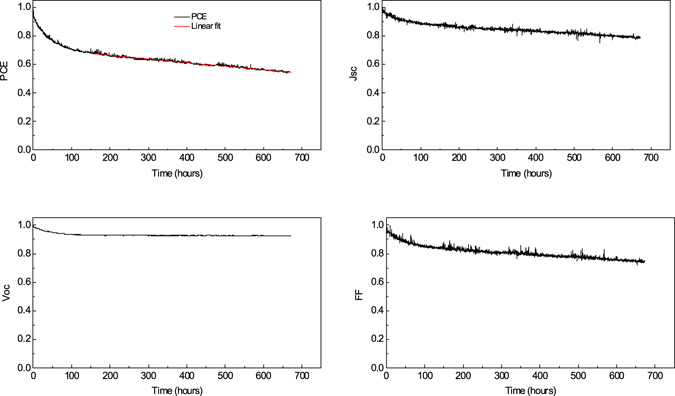
Evolution of the device metrics on testing using the indoor accelerated testing system normalised to initial values as a function time.

To understand the effect of device aging under well-controlled conditions, we have performed lifetime testing studies on a second series of nominally identical PFDT2BT-8:PC_71_BM OPV devices. These were performed under accelerated indoor conditions using the ATLAS Suntest solar simulator as shown in Fig. 5. As was observed in the outdoor testing experiment, device degradation is characterised by a rapid, initial burn-in process lasting approximately 150 hours in which PCE dropped by around 33% compared to its initial value. After this process, the reduction in PCE slowed and remained approximately constant for the rest of the experiment. We note that this loss in efficiency during the burn-in process is comparable with the value observed in outdoor tests. In contrast to the outdoor testing measurements, it can be seen that without climate fluctuations, the identification of the Ts80 lifetime is more straightforward. Here, we make a linear fit to device PCE over the period post burn-in, allowing us to determine a Ts80 lifetime of (530 ± 40) hours. In this case, the PCE degradation post burn-in resulted from linear reductions in both *J*_*sc*_ and *FF* with the *V*_*oc*_ again being relatively stable.

**Figure 6:**
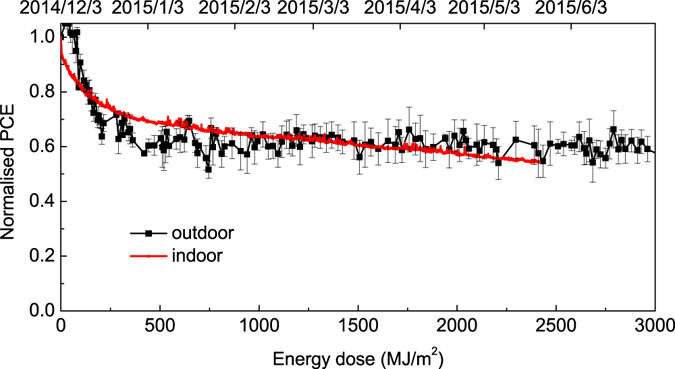
Comparison of outdoor/indoor efficiency degradation as a function of energy dose.

To explore the device degradation processes, Fig. 6 plots device PCE obtained from outdoor and indoor testing measurements as a function of total absorbed optical energy dose. Here data recorded from the device located in the indoors testing system was recorded over ~650 hours, while the equivalent energy dose received by devices tested outdoors was delivered over an exposure time of ~5.5 months. For this reason, the efficiency data plotted in Fig. 6 recorded from the devices tested out in the outdoors system is a subset of the data plotted in Fig. 3. For completeness we also present key parameters extracted from these two experiments in Table 2. As it can be seen, under both sets of testing conditions, the burn-in process ended when the devices received ~250 MJ/m^2^. This suggests that the burn-in process is associated with a photo-induced reaction within the active layer or at one or both interfaces that creates sub-bandgap states. Such states enhance energetic disorder reducing charge carrier mobility and also increase trap-mediated recombination, with both processes reducing device performance. Work on PCDTBT:PC_71_BM OPVs containing a controlled molecular weight distribution has also suggested that low molecular chain segments introduce deep traps in the active layer that trap charge and facilitate polymer oxidation, thus resulting in device burn-in ref. 40. We note that the common end point of the burn-in process observed under the two different testing conditions explored here suggests that this process is likely to be terminated by the depletion of some reaction species and that there is no apparent irradiance-level threshold to activate this reaction process^44^.

**Table 2:** A summary of key parameters determined from indoor and outdoor lifetime testing experiments.

Parameters	Burn-in time	Burn-in energy dose	Relative PCE after burn-in	Ts80	Energy dose at Ts80
Outdoor	1450 h	470 MJ/m^2^	63%	10430 h	2600 MJ/m^2^
Indoor	150 h	500 MJ/m^2^	68%	530 h	2000 MJ/m^2^

In the time-period post burn-in, it can be seen that there is a reasonable agreement between the two rates of device degradation, however devices tested using the accelerated indoor test system appear to reduce in efficiency more rapidly than devices tested outdoors. We speculate that this difference most likely results from the fact that the devices tested outdoors had a *second*-*level encapsulation consisting of a metal chamber filled with dry nitrogen*, while the devices tested indoors using both the Newport and ATLAS Suntest solar simulators *were simply encapsulated by glass and UV*-*cured epoxy*. This reduced level of encapsulation may have resulted in a slow ingress of oxygen and water to the device active layer that participated in the photo-generated oxidation of the active-layer^50, 51^. Note that using our current testing system we were not able to test OPV devices inside a metal test chamber in the indoor ATLAS Suntest solar simulator. This is because we found that if the test chambers were placed inside the solar simulator, the temperature within the chamber rapidly reached 105 °C. This elevated temperature would almost certainly generate additional degradation mechanisms resulting from thermally-driven morphological changes in the polymer:fullerene active layer. Indeed, previous work has shown that the degradation of P3HT:PCBM blend films is accelerated by elevated temperatures^52^. Despite such limitations, our experiments indicate that under the specific conditions explored here (i.e. different light levels and operating temperatures) the total optical radiation dose absorbed is correlated with operational device lifetime.

Finally, we note that the stability of the PFDT2BT-8:PC_71_BM devices studied here show some differences to our previous studies on OPV devices based on a different carbazole co-polymer (PCDTBT), in which Ts80 lifetimes (extrapolated) of up 14,500 hours were determined using extended exposure to light from an AM1.5 solar simulator^8^. The increased sensitivity observed here on the basis of indoor tests apparently results from a relatively accelerated reduction in efficiency in the period post burn-in. Nevertheless, in comparative outdoor studies, the PFDT2BT-8:PC_71_BM devices have apparently enhanced stability compared to devices based on PCDTBT-:PC_71_BM, in which Ts80 lifetimes of up to 6,200 hours were determined. We suspect that further improvements in device encapsulation may bring degradation rates measured between the different materials and data-sets presented in Fig. 6 into better agreement.

In summary, we have carried out the lifetime testing of OPV devices based on the donor-acceptor polymer:fullerene blend of PFDT2BT-8:PC_71_BM. We have used both outdoor and indoor testing techniques, with indoor tests following ISOS-L-1 standards, and outdoor measurements performed over a period of 12,000 hours (1.4 years). After 1.4 years, devices retain ~50% of their starting efficiency and have a PCE of (3.1 ± 0.1)% (Ts80 of 420 days). This level of stability using a relatively high efficiency donor-acceptor co-polymer is commensurate with some of the most stable devices reported based on the polymer P3HT^28^. This level of stability is achieved as devices were placed in a nitrogen-filled metal can having a glass lid and were protected from the atmosphere. It would of course be interesting to determine device stability when protected using more practical encapsulation schemes^27^, and in locations having a warmer climate as this may well result in accelerated degradation due to enhanced ingress of moisture into the device^25^. By comparing devices tested outdoors with lab-based measurements using an AM1.5 solar simulator, we show that using our specific experimental methodology, the device operational lifetime of the devices is approximately dependent on the total absorbed optical radiation dose.

## Methods

### Device fabrication

The PFDT2BT-8 used in these experiments was synthesised and purified according to previous methods^41^. Devices were fabricated on glass substrates coated with a patterned ITO electrode. Before processing, ITO substrates were cleaned in an ultrasonic bath using a Hellmanex solution, followed by cleaning in 2-propanol and deionised water. A PEDOT:PSS hole transport layer was then spin coated onto the substrates to create a film having a thickness of about 30 nm. After annealing at 120 °C for 5 minutes, substrates were transferred to a nitrogen-filled glove box. A solution of PFDT2BT-8 and PC_71_BM was then prepared at a mass ratio of 1:4 dissolved in chloroform at a total concentration of 20 mg/ml. The PFDT2BT-8:PC_71_BM solution was spin coated onto the PEDOT:PSS/ITO to create an active layer having a thickness of about 70 nm. A cathode comprising of 5 nm calcium and 100 nm aluminium was then thermally evaporated onto the active layer through a shadow mask under a vacuum of 2 × 10^−6^ mBar. This created 6 independent devices, each having an active area of 4 mm^2^. Finally, devices were encapsulated inside the glove-box using a glass cover-slip and UV-curable epoxy.

## Electronic supplementary material

Supplementary information for
