# Supplementary material for: Comparative indoor and outdoor stability measurements of polymer based solar cells
Source: Sci Rep. 2017 May 2;7:1305. doi: 10.1038/s41598-017-01505-w (PMC5431063; doi:10.1038/s41598-017-01505-w)
Supplement: Supplementary file 1 — Supplementary information for [file 41598_2017_1505_MOESM1_ESM.pdf]

Supplementary Information: Comparative indoor and outdoor stability measurements of polymer based solar cells

Yiwei Zhang[1], Hunan Yi[2], Ahmed Iraqi[2], James Kingsley[3],

Alastair Buckley[1], Tao Wang[4] and David G. Lidzey[1]*

[1]Department of Physics and Astronomy, University of Sheffield, Sheffield, S3 7RH, UK

[2]Department of Chemistry, University of Sheffield, Sheffield S3 7HF, UK

[3]Ossila Ltd, Kroto Innovation Centre, Broad Lane, Sheffield, S3 7HQ, UK

[4]School of Materials Science and Engineering, Wuhan University of Technology, Wuhan, 430070, China

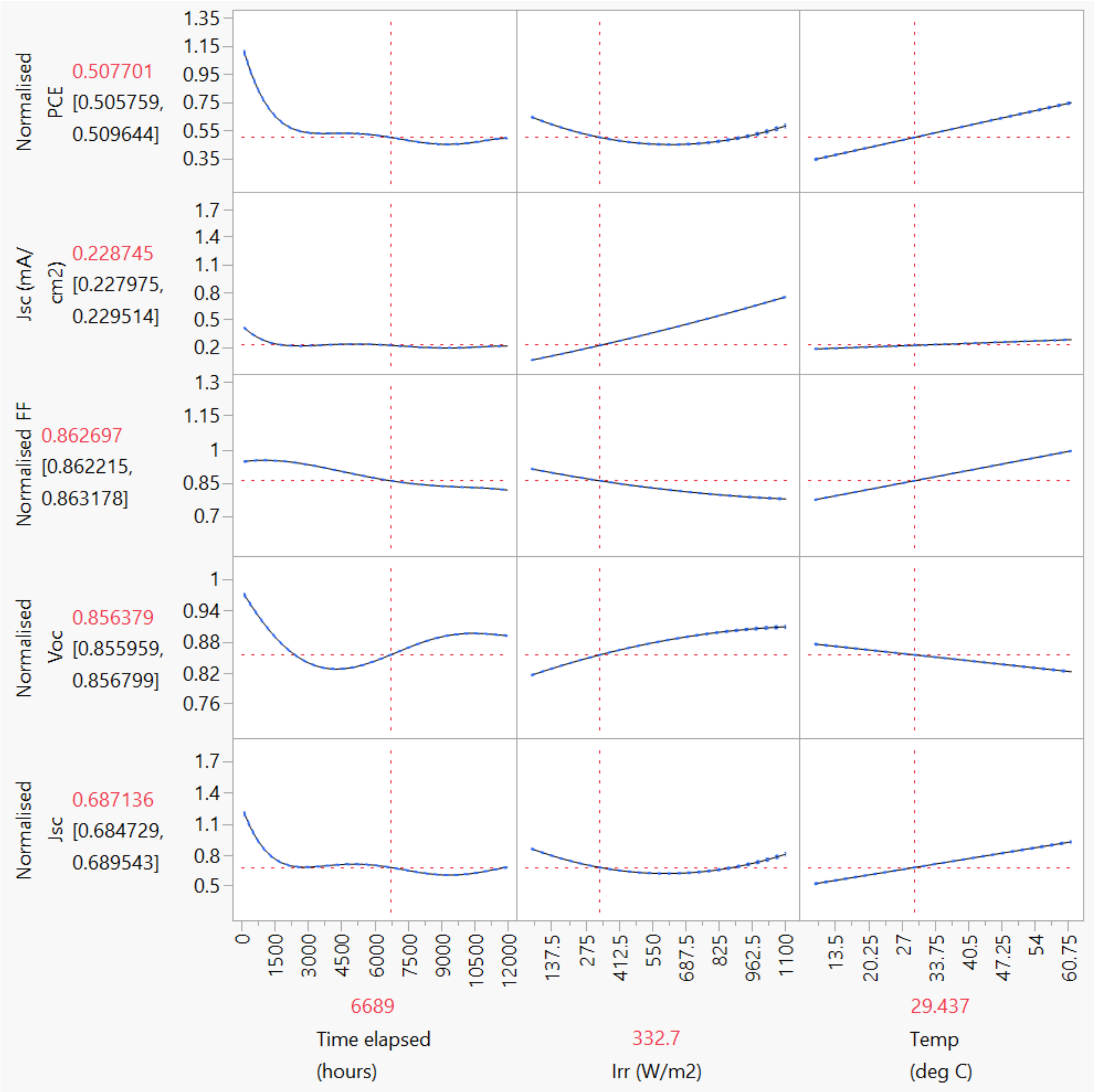

This plot shows the fitted model. It's not possible to overlap the experimental data on this plot since the functions shown are only cross sections through the multidimensional model

Time up to 5th order

Temp 1st order

Irr 2nd order

Irr x temp

Irr x time

Temp x time

Irr x time

R^2 for PCE fit = 0.5 (50% of variance in efficiency is explained)

R^2 for Jsc fit = 0.97 (97% of variance in Jsc is explained)

It is possible to compare the model for the different parameters in time. Efficiency and Jsc have been chosen. PCE shows the overall trend of how the devices degrade over time with 1st order effects of Irradiance and temperature taken out since Power output depends strongly on irradiance. Temperature effects are two orders of magnitude smaller but are still 1st order on power.

Plotting Jsc versus time allows these 1st order effects to be shown.

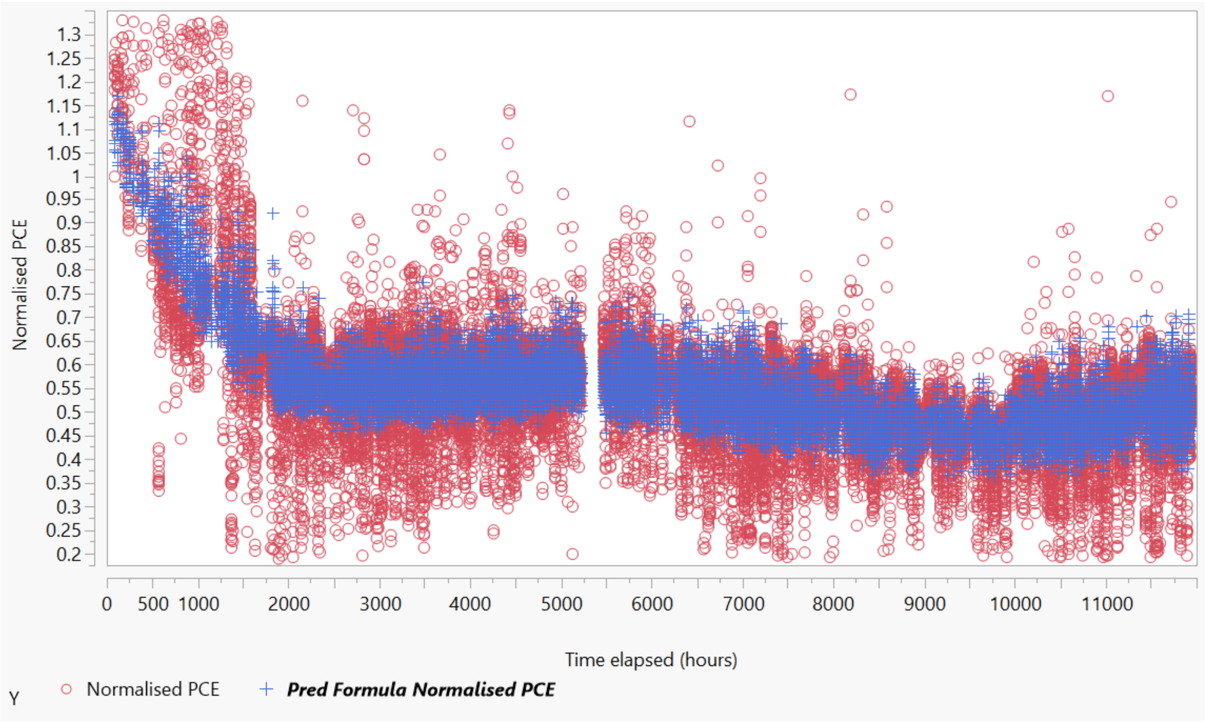

Measured (red) versus fitted model (blue)for PCE

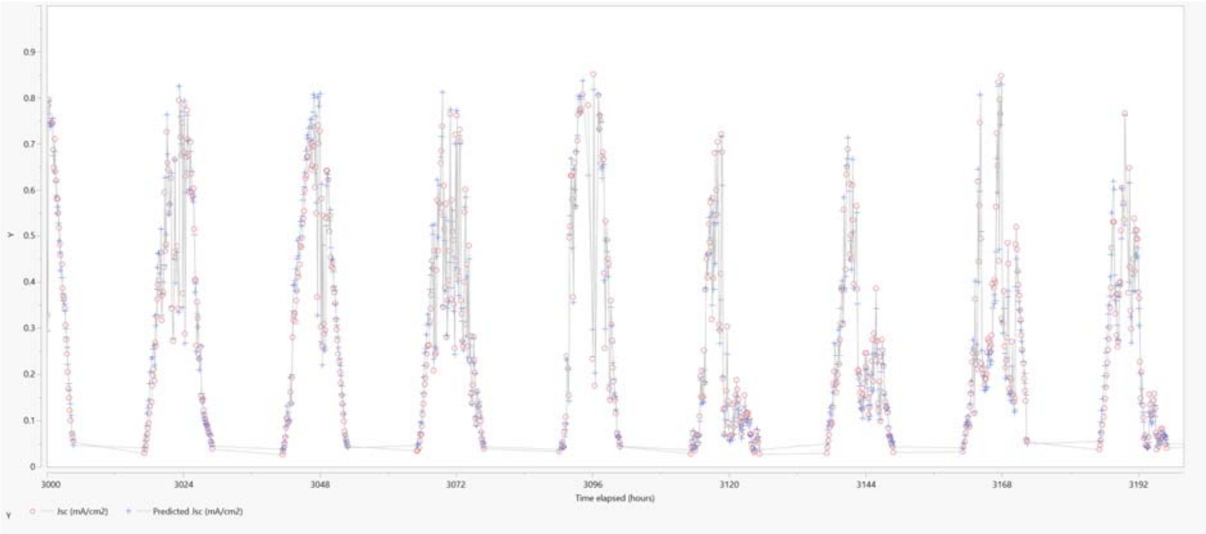

Measured (red) versus fitted model (blue) for Jsc for 8 day period.

To investigate whether there is any recovery overnight we fitted a further model including both time of day and morning or afternoon as factors in the model

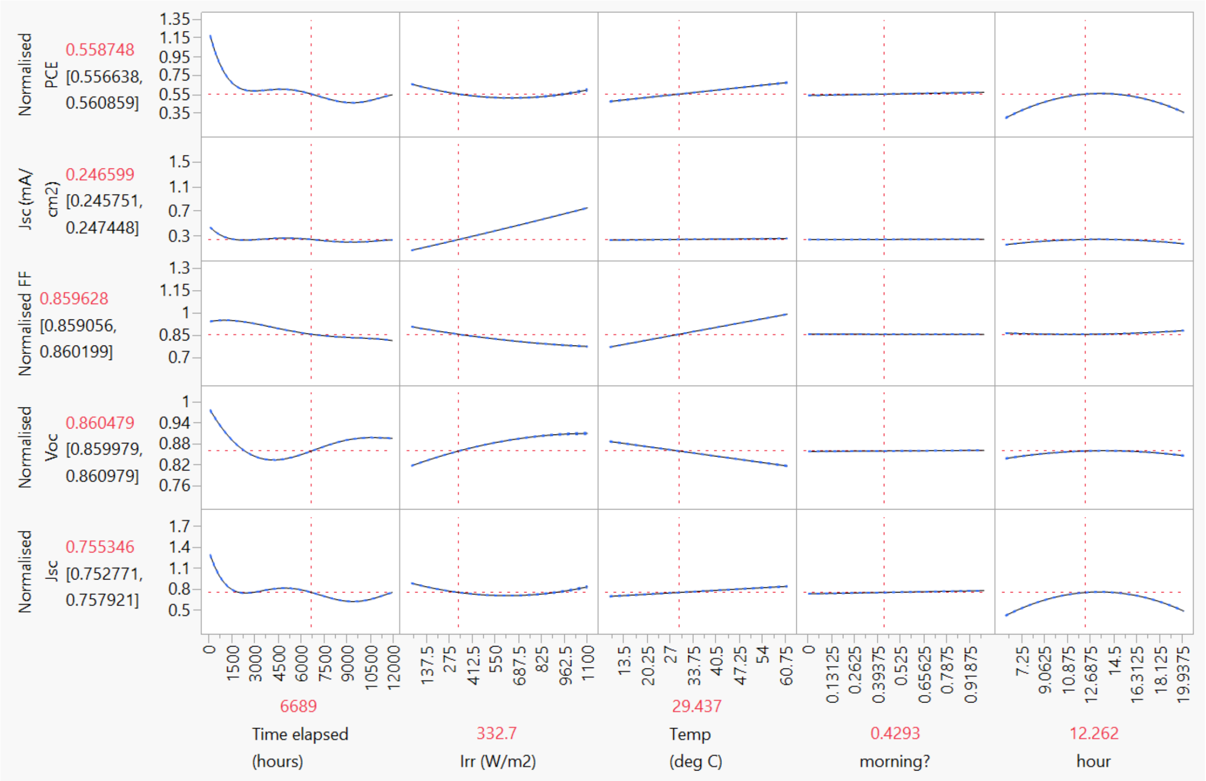

We find that the overall R^2 for PCE increases to 0.6 and that there is a slight improvement in performance in the afternoon. This is not consistent with overnight recovery but actually implies the contrary – that extended continuous operation favours performance.
